# “Stomach mass” as the first manifestation of IgG4-related disease: a case report

**DOI:** 10.1186/s12876-021-02013-y

**Published:** 2021-11-24

**Authors:** Fulong Zhang, Jing Xu, Yuandong Zhu, Qianneng Wu, Xincheng Xie, Yan Shi

**Affiliations:** Department of Gastroenterology, HangZhou Xixi Hospital, 2 Hengbu Street, Xihu District, HangZhou, 310032 China

**Keywords:** IgG4-related disease, Stomach tumour, First manifestation

## Abstract

**Background:**

IgG4-related disease mainly manifests as organomegaly and is accompanied by tissue fibrosis (Mimori, Mod Rheumatol 29(2):213, 2019) which is frequently confused with tumour (Dawei et al., J Gastroenterol Hepatol 29(12):1375–8, 2020). There are few reports with of IgG4-related disease with the first clinical manifestation involving the stomach.

**Case presentation:**

We present the case of 46-year-old male patient with a “stomach tumour” as the first manifestation of IgG4-related disease. Gastroscopy showed a mass in the stomach, however, the pathology result was chronic inflammation with IgG4 positivity. CT scans of abdomen showed that the stomach wall was thick, the head of the pancreas was swollen, and retroperitoneal fibrosis was severe.The serum IgG4 level was 75 g/L (normal range 0.03–2.01 g/L).After treatment with methylprednisolone for one month, the symptoms were greatly relieved.

**Conclusions:**

To reduce the suffering of patients and relieve their financial burden, we should consider the possibility of IgG4-related disease when the initial manifestation is a stomach mass.

## Background

IgG4-related disease is immune-mediated chronic inflammation that is diagnosed by clinicopathological presentations of enlarged organs, elevation of serum concentrations of IgG4 and pathological findings such as a typical dense lymphoplasmacytic infiltration enriched in IgG4-positive plasma cells, storiform fibrosis, and obliterative phlebitis [1, 2]. Pancreas, kidney, and retroperitoneum are often affected [3]. However, there are few reports of IgG4-related disease involving the stomach.This article analysed a patient with IgG4-related disease who had a “stomach tumor” as the initial manifestation.

## Case presentation

**Fig. 1:**
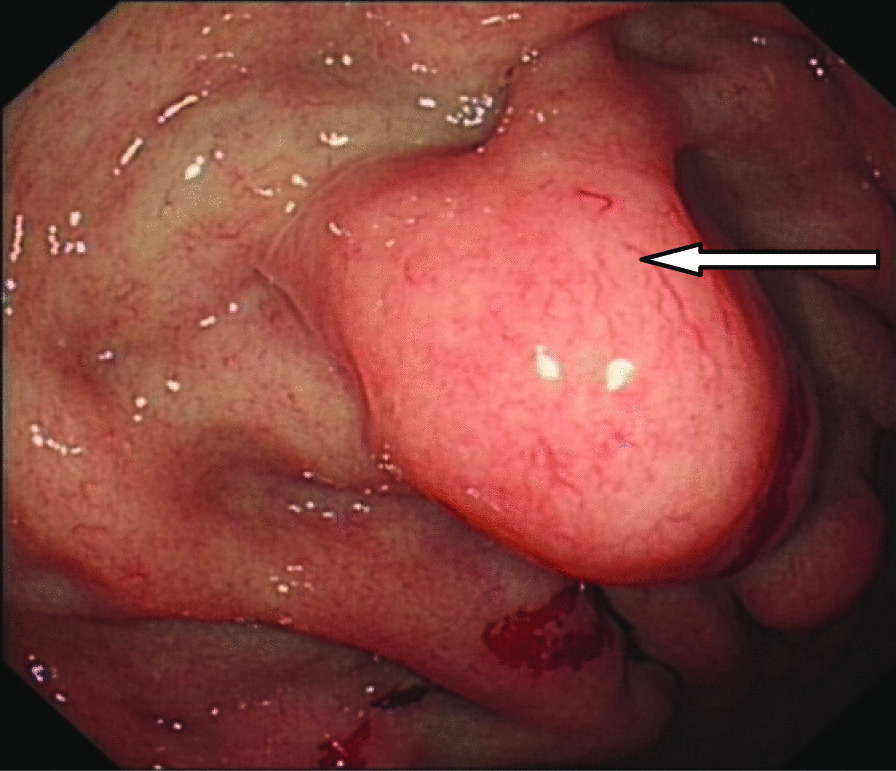
Stomach mass in gastroscopy before the treatment

**Fig. 2:**
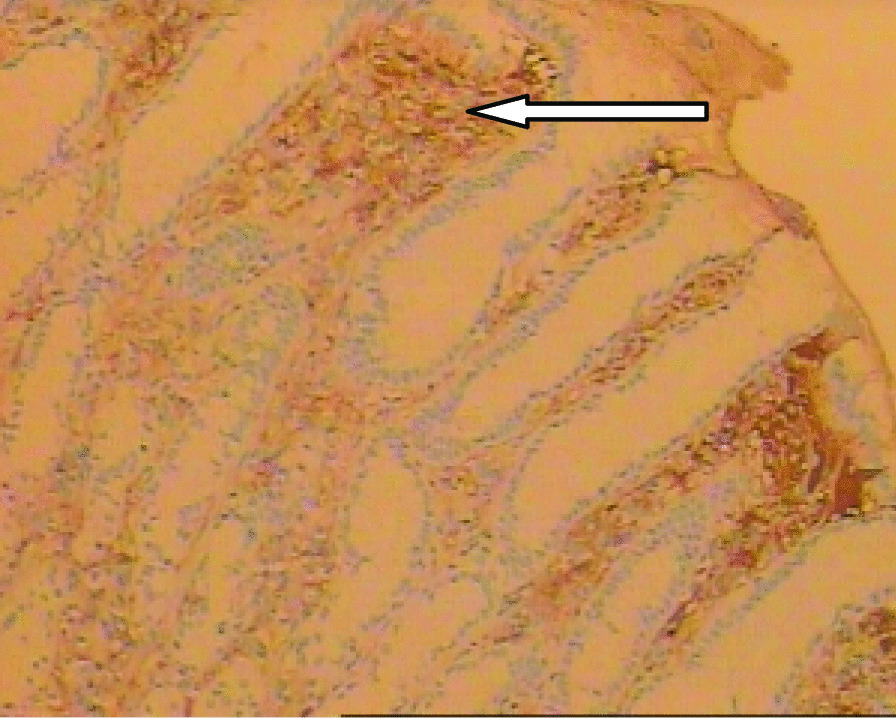
Gastric mucosa plasmacyte IgG4 positivity with immunohistochemistry

**Fig. 3:**
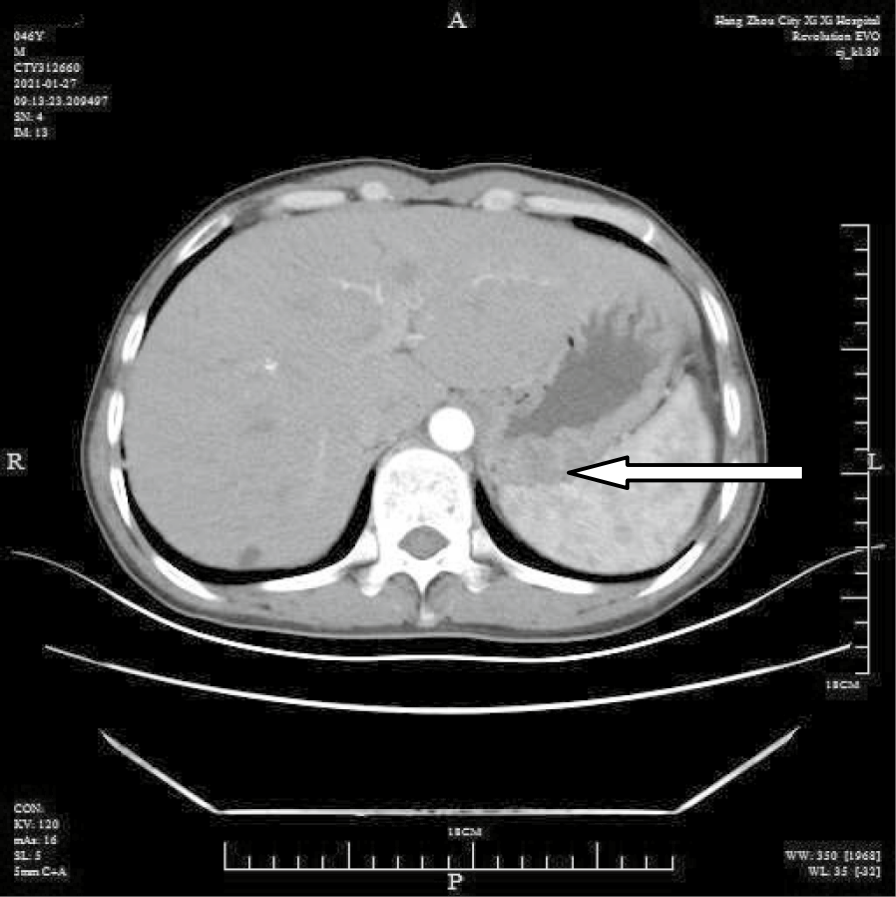
Thick stomach in CT before the treatment

**Fig. 4:**
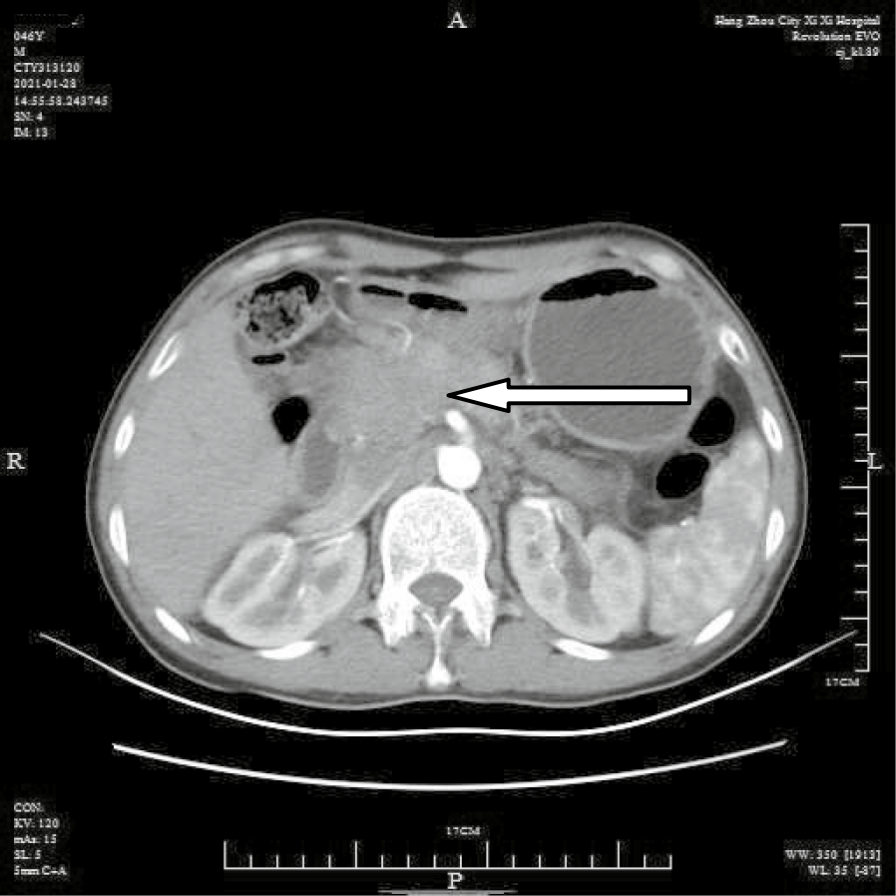
Head pancreas swollen in CT before the treatment

**Fig. 5:**
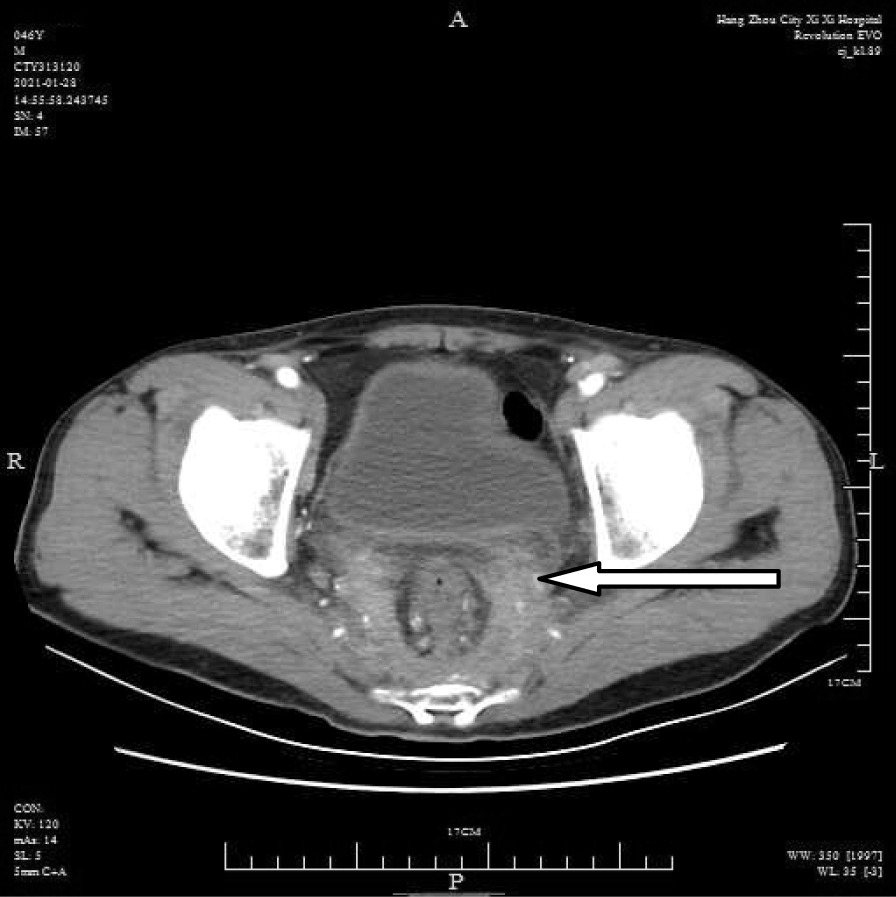
Retroperitoneal fibrosis in CT before the treatment

**Fig. 6:**
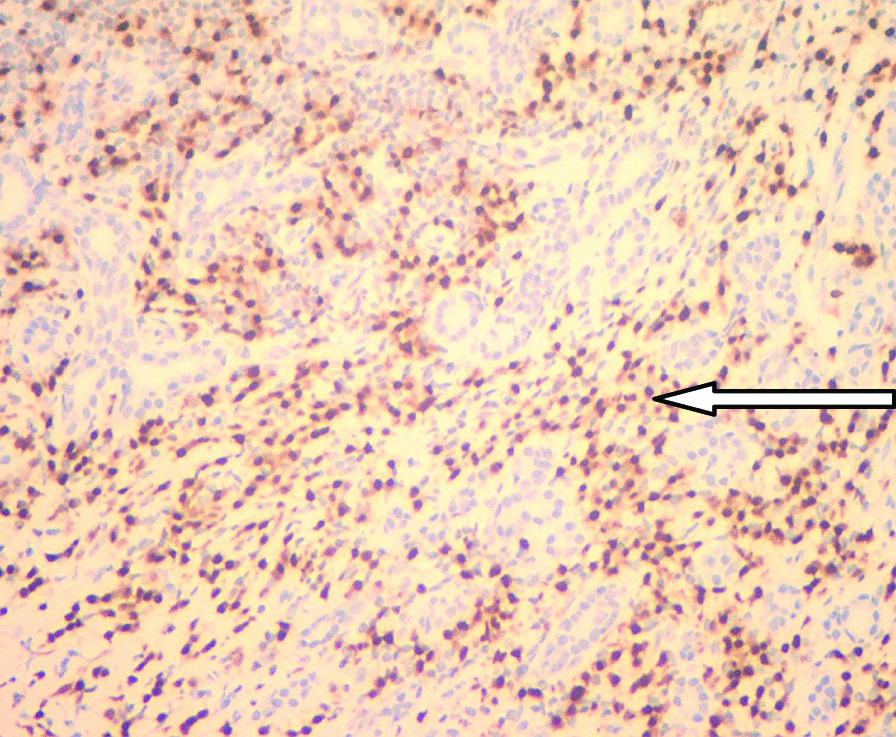
Right parotid gland plasmacyte IgG4 positivity with immunohistochemistry

**Fig. 7:**
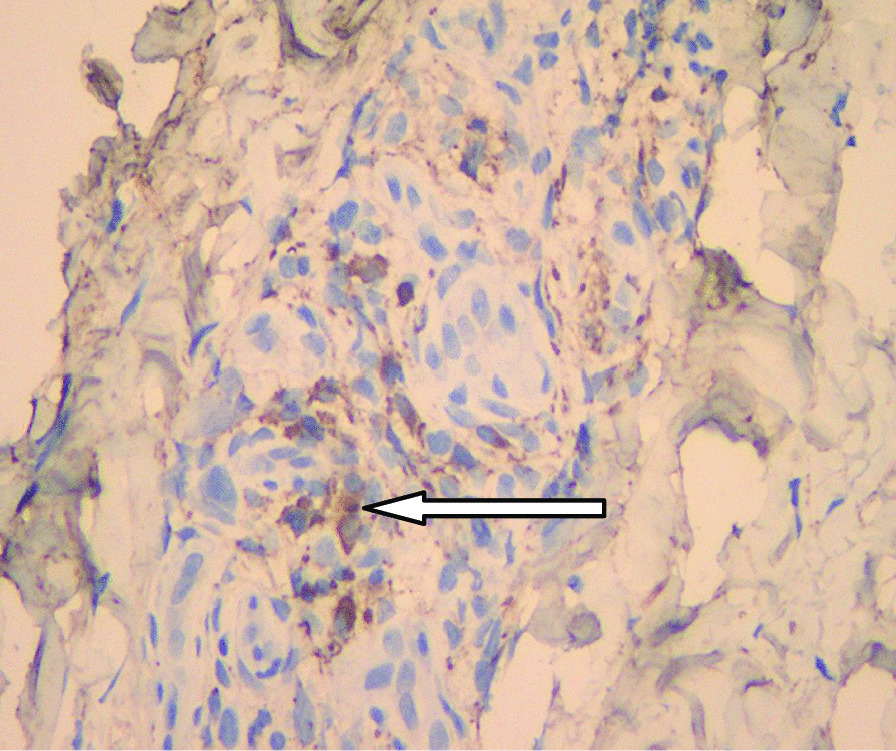
Left anterior tibia skin plasmacyte IgG4 positivity with immunohistochemistry

**Fig. 8:**
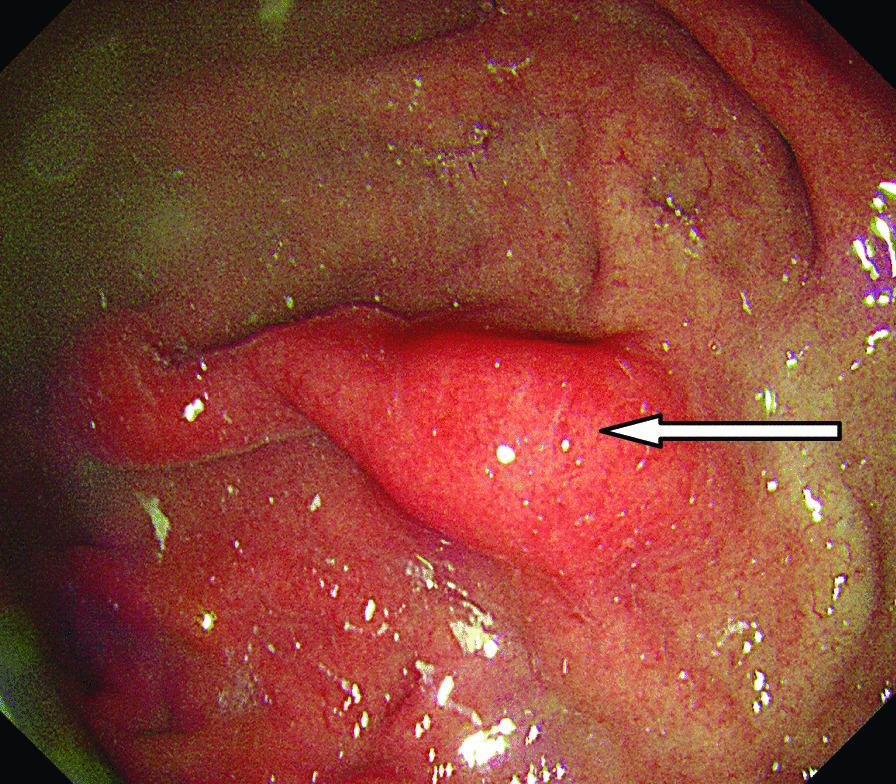
Stomach swollen in gastroscopy after one month treatment

**Fig. 9:**
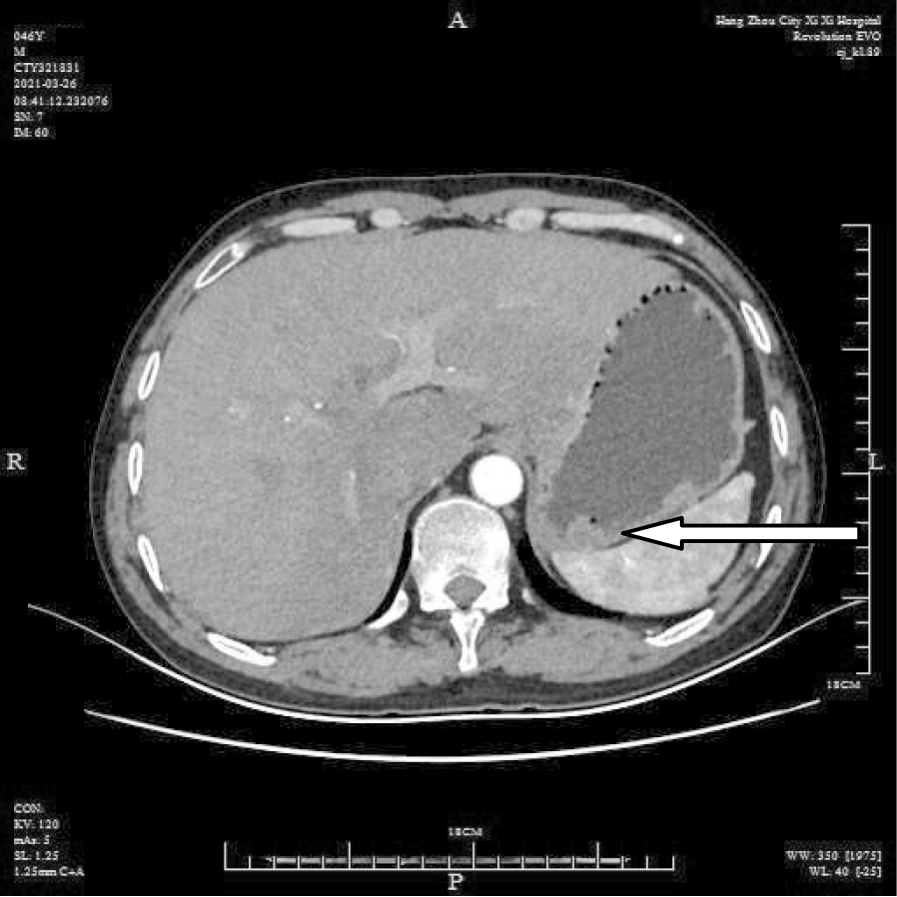
Slightly thickened stomach wall in CT after one month treatment

**Fig. 10:**
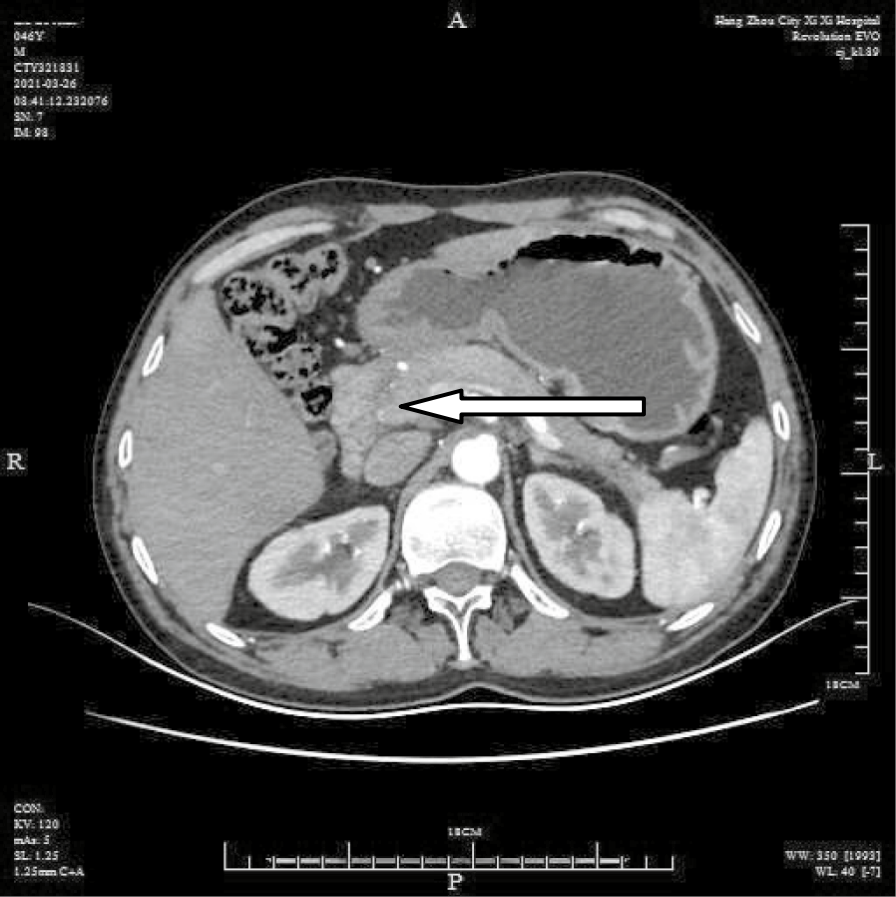
Head pancreas normal in CT after one month treatment

**Fig. 11:**
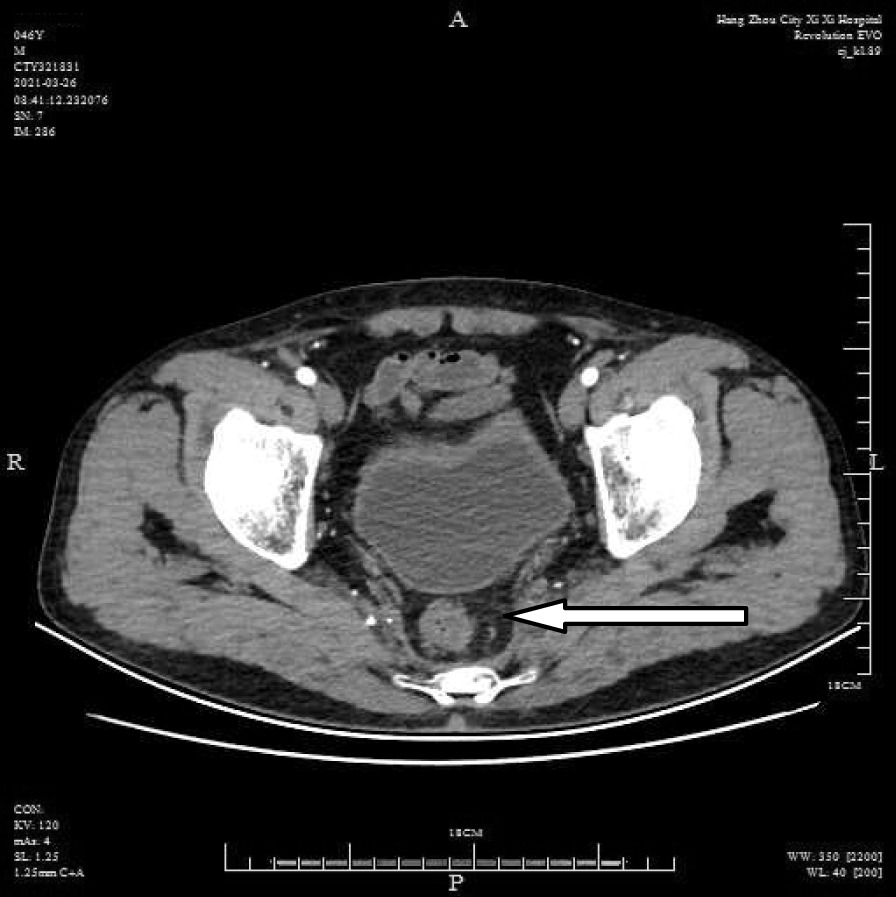
Retroperitoneum normal in CT after one month treatment

A 46-year-old male patient came to the hospital with discomfort of stomach for half a year and 10 kg weight loss. The patient had no long-term history of heavy alcohol or smoking consumption, and he had no history of hypertension, diabetes, coronary heart disease, or hepatitis, or allergic diseases. The patient had no corresponding family history. Physical examination revealed pigmentation on left anterior tibia skin and swelling of right parotid gland. Patient’s body mass index (BMI) was 18.32 kg/m^2^. Gastroscopy showed a mass in the stomach (Fig. 1), and the pathology result of the stomach mass biopsy was chronic inflammation with plasmacyte IgG4 positivity (Fig. 2).CT scans of the abdomen showed that the stomach wall was notably thick (Fig. 3), the head of the pancreas was swollen (Fig. 4), and retroperitoneal fibrosis was severe (Fig. 5). The serum IgG4 level was 75 g/L (normal range 0.03–2.01 g/L). The laboratory result showed hemoglobin (Hb), albumin (ALB), alanine aminotransferase (ALT), aspartate aminotransferase (AST), alkaline phosphatase (AKP), eosinophil (EO) were not in normal range (Table 1).Hepatitis B surface antigen and hepatitis A antibody, hepatitis C antibody, hepatitis E antibody, human immunodefciency virus antibody, anti-nuclear anti-bodies (ANA), anti-smooth muscle antibodies, anti-liver and kidney microsome antibodies, anti-mitochondrial antibodies, anti-myocardial antibodies, anti-parietal cell antibodies and anti-neutrophil cytoplasmic antibodies were all negative. T-spot, erythrocyte sedimentation rate (ESR), C-reactive protein (CRP), prothrombin time, D-dimer, white blood cell (WBC), platelet, lymphocyte were all normal. Later, biopsies of the right parotid gland (Fig. 6) and left anterior tibia skin (Fig. 7) were performed. The pathology results showed that tissue fibrosis was obvious in parotid gland and skin. Immunohistochemistry showed more than 50 IgG4-positive plasma cells/HPF. Methylprednisolone (40 mg) was given orally once a day for 2 weeks,and then 32 mg were given orally once a day for the next 2 weeks. After treatment, the patient’s stomach symptoms improved significantly and he gained weight (7.5 kg).Gastroscopy showed that the stomach mass was smaller than before methylprednisolone treatment (Fig. 8), and abdomeninal CT scans showed that the stomach wall was thin (Fig. 9),the head of the pancreas was normal (Fig. 10),and the retroperitoneal fibrosis was significantly reduced (Fig. 11).The serum IgG4 level decreased to 6.55 g/L.The HB, ALB, ALT, AST, AKP and EO level become nomal.

**Table 1:** The laboratory result of the patient before treatment

	Laboratory result
IgG4 (g/L)	75 (0.03–2.01)
ALB (g/L)	26.9 (40.0–55.0)
ALT (u/L)	89 (9–50)
AST (u/L)	104 (15–40)
AKP (u/L)	694 (301–120)
ESR (mm/h)	8 (0–15)
CRP (mg/L)	2.7 (0–10)
WBC (10^9^/L)	4.54 (3.4–9.5)
HB (g/L)	100 (130–175)
EO (10^9^/L)	0.97 (0.02–0.52)
Lymphocyte (10^9^/L)	3.1 (1.1–3.2)
Platelet (10^9^/L)	274 (125–350)
T-Spot (pg/ml)	3 (2.0–14.0)
Prothrombin time (S)	10.6 (9.7–13.5)
Dimer (mg/L)	0.09 (0.00–0.55)
ANA	Negative
Hepatitis B surface Antigen	Negative
Hepatitis A antibody	Negative
Hepatitis C antibody	Negative
Hepatitis E antibody	Negative
Human immunodefciency virus antibody	Negative
Anti-smooth muscle antibodies	Negative
Anti-liver and kidney microsome antibodies	Negative
Anti-mitochondrial antibodies	Negative
Anti-myocardial antibodies	Negative
Anti-parietal cell antibodies	Negative
Anti-neutrophil cytoplasmic antibodies	Negative

## Discussion and conclusions

The reason for IgG4-related disease is unknown, and the pancreas, biliary tract, lymph nodes, lungs, livers, pleura, kidneys and other organs can been involved [4]. When the digestive tract is involved, the symptoms are often complicated. Lopes [5] and Dumas-Campagna [6] reported that IgG4-related disease involving the digestive tract was diagnosed from the symptoms persent for 6 to 10 years. The manifestations of IgG4-related diseases involving the stomach is ulcers with mild inflammation of the surrounding mucosa [7]. In this case, IgG4-related disease involving the stomach was diagnosed from the symptom present for half a year,and the manifestation was bulge hyperplasia under gastroscopy. We referred to the diagnostic criteria of IgG4 in Japan in 2011 [8]: (1) diffuse or characteristic swelling and hypertrophy of single or multiple organs in clinic; (2) elevation of serum IgG4 ≥ 1.35 g/L; (3) significant in histopathological Infiltration and fibrosis of lymphocytes, with an IgG4 positive rate > 40% and > 10 IgG4-positive plasma cells/HPF. This patient met all three criteria and was diagnosed with IgG4-related disease. Zheng [9] reported that the infection rate of Helicobacter pylori in 10 cases of IgG4-related gastric disease was 20% (2/10). Helicobacter pylori was not infected in this case, and whether the level of IgG4 has an antagonistic effect on Helicobacter pylori needs further study. The treatment for IgG4-related disease is based on glucocorticoids, and prednisone 30–40 mg/d orally can induce remission [10]. After treatment with methylprednisolone for 1 month, significant organ improvement was observed. However, IgG4 disease easily recurs and can be aggravated by single glucocorticoid therapy [11]. Therefore, the therapy of this patient should be adjusted according to the patient’s condition.

When IgG4-related disease involves the stomach, the swelling of the stomach is similar to that of a tumour. The results of gastric mucosa biopsy, serum IgG4 level, and organomegaly or tissue fibrosis must be combined to confirm the diagnosis of IgG4-related disease. However, the prospective cohort study showed that Chinese IgG4 disease patients had a 2.78-fold increased risk of having malignancy, and the most common malignancy site was the gastrointestinal tract [12]. IgG4-related disease can create an immunological context that may impair the tumoral immunosurveillance and promote the pancreatic carcinogenesis [13]. Autoimmune pancreatitis was identifed as a potential risk factor for IgG4 related patients having malignancy [3]. Although autoimmune pancreatitis was not found in this patient, due to the potential for malignancy, physicians should be alert to this possibility during follow-up.

We should consider the possibility of IgG4-related disease and alert the potential for malignancy when a mass is seen in the stomach, to reduce the suffering for patients and the financial burden for their families.

## Data Availability

The datasets analysed during the current study are available from the corresponding author on reasonable request.

## Abbreviations

IgG: Immunoglobin G
CT: Computed tomography
HPF: High power field
BMI: Body mass index
Hb: Hemoglobin
ALB: Albumin
ALT: Alanine aminotransferase
AST: Aspartate aminotransferase
AKP: Alkalinephosphatase
EO: Eosinophil
ANA: Anti-nuclear anti-bodies
ESR: Erythrocyte sedimentation rate
CRP: C-reactive protein
WBC: White blood cell
